# A new hybrid record linkage process to make epidemiological databases interoperable: application to the GEMO and GENEPSO studies involving *BRCA1* and *BRCA2* mutation carriers

**DOI:** 10.1186/s12874-021-01299-6

**Published:** 2021-07-29

**Authors:** Yue Jiao, Fabienne Lesueur, Chloé-Agathe Azencott, Maïté Laurent, Noura Mebirouk, Lilian Laborde, Juana Beauvallet, Marie-Gabrielle Dondon, Séverine Eon-Marchais, Anthony Laugé, GEMO Study Collaborators, Nadia Boutry-Kryza, Alain Calender, Sophie Giraud, Mélanie Léone, Brigitte Bressac-de-Paillerets, Olivier Caron, Marine Guillaud-Bataille, Yves-Jean Bignon, Nancy Uhrhammer, Valérie Bonadona, Christine Lasset, Pascaline Berthet, Laurent Castera, Dominique Vaur, Violaine Bourdon, Catherine Noguès, Tetsuro Noguchi, Cornel Popovici, Audrey Remenieras, Hagay Sobol, Isabelle Coupier, Pierre-Olivier Harmand, Pascal Pujol, Paul Vilquin, Aurélie Dumont, Françoise Révillion, Danièle Muller, Emmanuelle Barouk-Simonet, Françoise Bonnet, Virginie Bubien, Michel Longy, Nicolas Sévenet, Laurence Gladieff, Rosine Guimbaud, Viviane Feillel, Christine Toulas, Hélène Dreyfus, Dominique Leroux, Magalie Peysselon, Christine Rebischung, Amandine Baurand, Geoffrey Bertolone, Fanny Coron, Laurence Faivre, Vincent Goussot, Caroline Jacquot, Caroline Sawka, Caroline Kientz, Marine Lebrun, Fabienne Prieur, Sandra Fert-Ferrer, Véronique Mari, Laurence Vénat-Bouvet, Stéphane Bézieau, Capucine Delnatte, Isabelle Mortemousque, Florence Coulet, Florent Soubrier, Mathilde Warcoin, Myriam Bronner, Sarab Lizard, Johanna Sokolowska, Marie-Agnès Collonge-Rame, Alexandre Damette, Paul Gesta, Hakima Lallaoui, Jean Chiesa, Denise Molina-Gomes, Olivier Ingster, Sylvie Manouvrier-Hanu, Sophie Lejeune, GENEPSO Study Collaborators, Catherine Noguès, Lilian Laborde, Pauline Pontois, Dominique Stoppa-Lyonnet, Marion Gauthier-Villars, Bruno Buecher, Olivier Caron, Emmanuelle Mouret-Fourme, Jean-Pierre Fricker, Christine Lasset, Valérie Bonadona, Pascaline Berthet, Laurence Faivre, Elisabeth Luporsi, Marc Frénay, Laurence Gladieff, Paul Gesta, Hagay Sobol, François Eisinger, Jessica Moretta, Michel Longy, Catherine Dugast, Chrystelle Colas, Florent Soubrier, Isabelle Coupier, Pascal Pujol, Alain Lortholary, Philippe Vennin, Claude Adenis, Tan Dat Nguyen, Capucine Delnatte, Annick Rossi, Julie Tinat, Isabelle Tennevet, Jean-Marc Limacher, Christine Maugard, Yves-Jean Bignon, Liliane Demange, Hélène Dreyfus, Odile Cohen-Haguenauer, Brigitte Gilbert, Dominique Leroux, Hélène Zattara-Cannoni, Catherine Noguès, Nadine Andrieu, Dominique Stoppa-Lyonnet, Sandrine M. Caputo

**Affiliations:** 1 Department of Genetics, Institut Curie, PSL Research University, Paris, France; 2 Inserm, U900, Paris, France; 3 Institut Curie, PSL Research University, Mines ParisTech, Paris, France; 4 Mines ParisTech, PSL Research University, CBIO-Centre for Computational Biology, Paris, France; 5 Institut Paoli-Calmettes, Centre de Traitement des Données IPC-PACA, Département de la Recherche Clinique et de l’Innovation, Marseille, France; 6 Institut Paoli-Calmettes, Département d’Anticipation et de Suivi du Cancer, Oncogénétique clinique, Marseille France Inserm, U830, Université Paris Descartes, Paris, France; 7 Aix Marseille Univ, INSERM, IRD, SESSTIM, Sciences Economiques et Sociales de la Santé & Traitement de l’Information Médicale, Marseille, France; 8 Paris University, Paris, France; 9 Inserm, U830, Paris, France

**Keywords:** Record linkage, Hybrid process, Probabilistic linkage, Supervised machine learning

## Abstract

**Background:**

Linking independent sources of data describing the same individuals enable innovative epidemiological and health studies but require a robust record linkage approach. We describe a hybrid record linkage process to link databases from two independent ongoing French national studies, GEMO (Genetic Modifiers of *BRCA1* and *BRCA2*), which focuses on the identification of genetic factors modifying cancer risk of *BRCA1* and *BRCA2* mutation carriers, and GENEPSO (prospective cohort of *BRCAx* mutation carriers), which focuses on environmental and lifestyle risk factors.

**Methods:**

To identify as many as possible of the individuals participating in the two studies but not registered by a shared identifier, we combined probabilistic record linkage (PRL) and supervised machine learning (ML). This approach (named “PRL + ML”) combined together the candidate matches identified by both approaches. We built the ML model using the gold standard on a first version of the two databases as a training dataset. This gold standard was obtained from PRL-derived matches verified by an exhaustive manual review. Results

The Random Forest (RF) algorithm showed a highest recall (0.985) among six widely used ML algorithms: RF, Bagged trees, AdaBoost, Support Vector Machine, Neural Network.

Therefore, RF was selected to build the ML model since our goal was to identify the maximum number of true matches. Our combined linkage PRL + ML showed a higher recall (range 0.988–0.992) than either PRL (range 0.916–0.991) or ML (0.981) alone. It identified 1995 individuals participating in both GEMO (6375 participants) and GENEPSO (4925 participants).

**Conclusions:**

Our hybrid linkage process represents an efficient tool for linking GEMO and GENEPSO. It may be generalizable to other epidemiological studies involving other databases and registries.

**Supplementary Information:**

The online version contains supplementary material available at 10.1186/s12874-021-01299-6.

## Background

Record linkage is a process that allows to identify records appearing in different databases and referring to the same entity (e.g. an individual) [1], but which do not share a common unique identifier. In record linkage, the status of a pair of records is either matching (same individual) or non-matching (distinct individuals). This process consists in three successive steps: data preprocessing (curation of the data), record pair comparison and linkage. Data preprocessing includes harmonizing data formats and dealing with missing values. The record pair comparison can be computationally expensive, as the number of all possible record pairs is the product of the numbers of records in each dataset. To reduce the number of comparisons to run, it is common to perform blocking. Blocking consists in splitting the datasets into smaller sets that agree on one or more variables, called blocking variables. Only records within the same blocks are then compared. When no unique person identifier is shared between the two datasets, linkage has to be performed by comparison of shared matching variables. The linkage performance is assessed by comparison with the gold standard (or ground truth) based on a confusion matrix [2]. The record linkage matches may have two types of errors: False Positives (FP), i.e. true non-matches classified as matches, and False Negatives (FN), i.e. true matches classified as non-matches.

Linkage methods are usually classified as either deterministic or probabilistic [1, 3, 4]. Deterministic record linkage methods assess matching status based on the exact agreement or disagreement of either all or a fraction of the matching variables. If data are of very good quality (i.e. no more than 5% of missing data or errors in any matching variable), the deterministic linkage can have a satisfying linkage quality. Otherwise, it will produce a large number of FNs [5]. By contrast, Probabilistic Record Linkage (PRL) aims at determining the probability that two records refer to the same individual. Rather than requesting exact agreement of the matching variables, PRL can use similarity scores between the values taken by the matching variables. PRL takes into account the difference in discriminatory power of each matching variable. Indeed, the more frequent a value of matching variable is, the less discriminative for linkage this value is. In practice, PRL can give better results than deterministic linkage when the data are not of good quality [6]. To allow for typing errors or spelling changes, the values, in two records, of a matching variable are compared using a similarity function, which returns a similarity score. These scores are used as input by linkage methods to classify record pairs into matches and non-matches. In PRL, the determination of the threshold on the likelihood scores that separate the matches from the non-matches is critical and has a direct impact on the relative numbers of FP and FN [7]. Although the threshold could be estimated by controlling the theoretical FP and FN rates [3], the most common practice is to examine the empirical distribution of scores, and chose the threshold according to a predefined FN or FN rate. From a machine learning point of view, record linkage can be considered as a classification task. Each record pair is represented by a comparison vector containing, for each matching variable, the similarity score between both records. The supervised machine learning (ML) algorithm learns a model that takes such a comparison vector as input and returns matching status as output, based on a training set in which the matching status of record pairs are known. Various ML algorithms have been applied to record linkage, such as Classification Tree (CT), Support Vector Machines (SVM), Neural Networks (NNET), or Random Forest (RF) [8–13]. However, their application is usually limited by the need of a training set.

We therefore conduct a study, in which we learn an ML model from a training set where the ground truth was established by PRL followed by manual review. Because PRL and ML methods may make distinct errors [14], we also propose to combine PRL with the ML model we have trained. We applied this hybrid linkage process to match individuals between the GEMO (Genetic Modifiers of *BRCA1* and *BRCA2*) [15] and the GENEPSO (prospective cohort of *BRCAx* mutation carriers) [16] studies, building our PRL + ML approach on a first version of those studies, and applying it to their updated versions.

GEMO and GENEPSO are two independent ongoing nation-wide studies involving *BRCA1* and *BRCA2* carriers, with unconnected databases and whose individuals were not registered by a shared identifier. *BRCA1* and *BRCA2* genes testing has become part of routine clinical practice in European countries and North America since the identification of the two genes in the 90’s, which greatly improved recommendations about breast and ovarian cancer risk management treatments. Nonetheless, both retrospective and prospective studies on large datasets of *BRCA1* and *BRCA2 (BRCA1/2)* mutation carrier families are very much needed to refine individual cancer risk estimates by using different cancer risk factors such as genetic factors, lifestyle/environmental factors, family history and breast pathology.

GEMO and GENEPSO provide an overview of a well-characterized sample of counseled Hereditary Breast and Ovarian Cancer (HBOC) families in France. Through the GEMO study, blood DNA from *BRCA1/2* mutation carriers is available to perform genetic epidemiological projects aiming at identifying and characterizing genetic factors modifying breast and ovarian cancer risk. In the prospective cohort GENEPSO, which aims at assessing environmental and lifestyle risk factors, *BRCA1/2* mutation carriers are followed over time to observe characteristics of subjects who are developing either primary or secondary cancer.

GEMO and GENEPSO were set up at different time by two different coordinating centers and investigators involved in the Genetics and Cancer Group (GCG, UNICANCER) [17], a French multicenter group composed of clinicians, molecular geneticists and scientists. Participants in both studies undergo genetic counseling and they are invited to participate in GEMO and/or GENEPSO through the family cancer clinics if tested positive for a mutation in *BRCA1* or *BRCA2*. About 26% of index cases carrying such a mutation (i.e. the first individual tested in the family) are included in GEMO, and 21% in GENEPSO [18]. Therefore, it is essential to identify the overlap between participants of the both studies by linking the two data sources, which will allow setting up studies evaluating simultaneously genetic and non-genetic factors modifying cancer risk of carriers of a *BRCA1* or *BRCA2* mutation. Studies conducted in subjects enrolled in both studies will also allow, for instance, assessment of whether it is possible to predict response to treatment according to *BRCA1/2* mutation status and other genetic variant profile.

## Methods

### Data

In September 2016, 4688 participants had been enrolled in GEMO and 3339 in GENEPSO. This initial dataset (dataset 1) was used for building ML algorithms and determining an optimal linkage method. This optimal linkage method was then applied on the updated version (dataset 2) of the two studies as of December 2019. The updated version counted 6375 participants for GEMO (i.e. 1687 new participants), and 4925 participants for GENEPSO (i.e. 1586 new participants).

Name and address of individuals were not available here due to privacy and confidentiality policies. Ten matching variables shared between GEMO and GENEPSO were used for comparison (Table 1): recruiting center number (CTR), family number (NUMFAM), individual number in the family (SUJID), gender (GENDER), year of birth (Yob), month of birth (Mob), day of birth (Dob), *BRCA1* mutational status (BRCA1), *BRCA2* mutational status (BRCA2) and mutation description using the HGVS nomenclature (MUT_HGVS). Recruiting centers may be coded differently in GEMO and GENEPSO, however we were able to standardize the CTR variable during data pre-processing (see next paragraph). NUMFAM and SUJID are assigned by the recruiting center at the time of recruitment. In principle, same family number and individual number should be provided by the clinician to GEMO and GENEPSO investigators. However, the family number may be recorded under different formats in the two databases. Moreover, an individual may be assigned a different individual number on the pedigree (SUJID) in GEMO and in GENEPSO, if included at a different time in the two studies. These inconsistencies may be corrected later by manual review if other variables such as recruiting center, sex, *BRCA* mutation (HGVS nomenclature) and date of birth are identical in the two databases. The mutation description (MUT_HGVS) is a good matching variable as *BRCA1/2* mutations are rare and diverse [15, 19]. *BRCA1/BRCA2* mutation descriptions should theoretically use the international HGVS nomenclature in both database [20]. However, the two studies were independently set up more than 20 years ago and standards for mutation descriptions have evolved during these years. Therefore, the same mutation identified at different times by two laboratories may have been recorded with a different nomenclature. We thus had to standardize this annotation during data pre-processing (see next paragraph).

**Table 1:** An example of a record pair comparison and its PRL likelihood score calculation

	CTR	NUMFAM	SUJID	GENDER	Yob	Mob	Dob	BRCA1	BRCA2	MUT_HGVS	PRL Score
Individual GEMO__5789_	1	17455	0001	2	1959	08	05	1	0	c.3403C > T	–
Individual GENEPSO__01082300001_	1	08230	0001	2	1958	08	05	1	0	c. 3481_3491del	–
Similarity ***s***	1	0	1	1	0	1	1	1	1	0.7825	–
*f*	0.02272	0.00025	0.0018	0.5000	0.01098	0.07692	0.03125	0.3333	0.3333	0.0006	–
*w*	5.45	11.95	9.1	0.99	6.49	3.68	4.99	1.57	1.57	10.69	sum(*w*) = 56.48
w*s	5.45	0	9.1	0.99	0	3.68	4.99	1.57	1.57	8.36	sum(*w* * *s*) = 35.71
score ***S***											0.6322

Ten matching variables were used to identify record pairs: *BRCA1* mutational status (BRCA1), *BRCA2* mutational status (BRCA2), mutation description using the HGVS nomenclature (MUT_HGVS), gender (GENDER), recruiting center number (CTR), family number (NUMFAM), individual number in the family (SUJID), year of birth (Yob), month of birth (Mob) and day of birth (Dob). BRCA1 and BRCA2 matching variable: 1: “carrier of a *BRCA1/2* mutation”, 0: “non-carrier of a *BRCA1/2* mutation”. GENDER matching variable: 1: male, 2: female. The similarity vector ***s*** in the third row is used as input in the machine learning approaches. The PRL score ***S*** is calculated from the weight ***w*** and the similarity ***s***

### Data pre-processing

Record linkage is highly sensitive to data quality. Therefore, we performed data cleaning and standardization [21–23], such as removing duplicates, deleting spaces in strings, standardizing the format of matching and blocking variables, converting mutation descriptions to standard Human Genome Variation Society (HGVS) nomenclature [24], standardizing recruiting center number (CTR), and splitting dates of birth into month, day and year in order to compare respectively each of them and give credit for partial agreement.

### Record pair comparison

Let *X* and *Y* be two databases and *x* ∈ *X* and *y* ∈ *Y* two arbitrary records in form of a *d*-dimensional vector, i.e. *x* = [*x*_1_, …*x*_*d*_] and *y* = [*y*_1_, …*y*_*d*_]. *d* is the number of matching variables; in our study, *d* = 10. The space of comparison is the Cartesian product *X* × *Y* which contains of all possible record pairs (*x*, *y*). All matching variables are discrete numerical values except MUT_HGVS which is a string. A similarity vector *s* = [*s*_1_, …, *s*_*d*_] is then computed as *s*_*i*_ = (*x*_*i*_, *y*_*i*_) where *x*_*i*_, *y*_*i*_ are the *i*-th matching variables and (·, ·) is a measure of similarity given by the Jaro-Winkler similarity *sim*_*JW*_ for the string matching variable (MUT_HGVS) and by the binary similarity *sim*_*B*_ (i.e. exact agreement) for the others (Supplementary Data). Because of the evolution in standards for mutation descriptions during the last twenty years, a same *BRCA* mutation may have been annotated differently at different time by different laboratories from database to database. Even though we standardized MUT_HGVS variable as much as possible during data preprocessing, the inconsistencies in reporting the same mutation in GEMO and GENEPSO still existed. This is why we allowed inexact matching for the HGVS_MUT variable.

### Probabilistic record linkage (PRL)

The probability of matching for record pair (*x*, *y*) is computed as a weighted sum of the similarity vector S:

1 $$ S\left(x,y\right)=\frac{\sum_i{w}_i\; sim\left({x}_i,{y}_i\right)}{\sum_i{w}_i} $$

where *w* = [*w*_1_, …*w*_*d*_] is the vector of weights. Weights are computed using the EpiLink approach [25]; more specifically, for matching variable i,

$$ {w}_i={\mathit{\log}}_2\frac{\left(1-{e}_i\right)}{f_i} $$

where *f*_*i*_ denotes the average frequency of values taken by the variable and *e*_*i*_ the estimated error rate. We assumed *e*_*i*_ = 0.01 for all matching variable [25]. Since most software packages implementing PRL were found to perform similarly [26], we used the RecordLinkage R package [27]. We calculated the PRL score based on the ten matching variables. An example is shown in Table 1.

### Supervised machine learning (ML) linkage

We first used blocking to reduce the number of possible record pair comparisons. Missing data in blocking variables (BRCA1, BRCA2, GENDER and Yob) were tolerated here. After blocking, the imputation of missing data could be then performed. The missing data in similarity for MUT_HGVS (numeric) were imputed by Bayesian linear regression and those for other categorical matching variables were imputed by logistic regression.

The labeled record pairs were randomly partitioned into two sets: the training dataset (60%) on which we trained ML models, and the test dataset (40%) on which we evaluated the predictive performance of the trained models. ML models were built by using the similarity vector of the six variables (CTR, NUMFAM, SUJID, MUT_HGVS, Mob, Dob). We employed six broadly used ML algorithms (CT, Bagged trees, AdaBoost, RF, SVM and NNET) (Supplementary Data). We compared the performance of these 6 algorithms to that of a naive baseline, consisting in a Bernoulli model that randomly classifies a record pair as matching or non-matching.

### Hybrid record linkage process

**Fig. 1:**
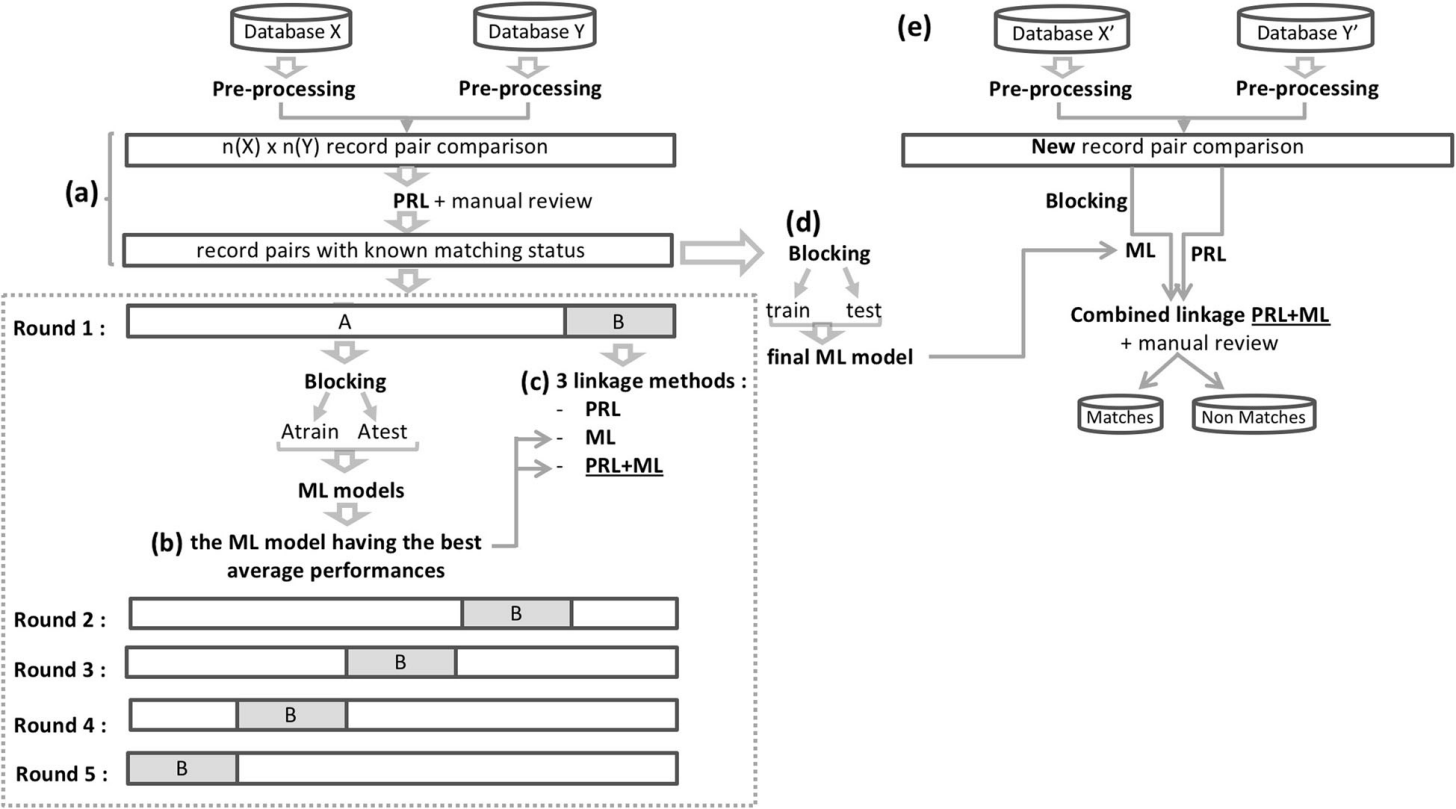
Elaboration of hybrid record linkage process and main steps. **a** Assignment the matching status by PRL followed by manual review, so that we could obtain a set of true matches representing the gold standard. **b** Selection of the best-performing supervised machine learning algorithm **c** Selection of the best-performing methods among PRL, ML, and PRL + ML **d** Training of a final ML model on a larger subset of initial datasets. **e** Application of the optimal linkage method to link the updated databases

Step 1 (Fig. 1a): after data pre-processing, we built record pair comparisons using dataset 1. We then computed a PRL score *S* from Eq. 1 for each pair. After examining the empirical distribution of PRL scores, we chose a threshold t to separate all record pairs into potential matches (*S*(*x*, *y*) > t) and non-matches (*S*(*x*, *y*) ≤ t). We manually reviewed not only these potential matches [28], but also the potential FNs. We launched several rounds of searches to find out the record pairs with PRL scores below the chosen threshold that may be FN. This exhaustive manual review was essential to minimize the number of FN, so that we could obtain a set of true matches representing the gold standard used to generate the confusion matrix of the dataset 1.

Step 2 (Fig. 1b, c, d): based on the gold standard of true matches from step 1, we selected the optimal linkage method by using a nested validation procedure. More specifically, we used a 5-fold cross-validation to compare the average performances of PRL, ML and PRL + ML. In the PRL + ML method, we first implemented PRL and ML in parallel, then combined together the candidate matches classified by both approaches.

Within the cross-validation, we used a 60–40% train-test split procedure to pick the ML algorithm having the best average performance. Within this procedure, we used a 5-fold inner cross-validation to determine the optimal parameters for each of the ML algorithms we investigated (See Supplementary Data for details).

We evaluated the performances of ML algorithms and the three linkage methods (PRL, ML, PRL + ML) based on the gold standard of true matches from step 1.

Once the best performing ML algorithm and the optimal linkage method was chosen, it is necessary to train a final ML model for the ML or PRL + ML linkage method to predict the new true matches on the updated dataset. To achieve this, after the blocking step and the imputation of missing data on the whole record pairs (from step 1), we trained a ML model on a larger subset than any “dataset A” used in the cross-validations step (Fig. 1b and c), and verified whether this model still had a good performance on the remaining subset.

Step 3 (Fig. 1e): we applied the selected linkage method followed by manual review to identify new true matches on the updated dataset.

### Performance measures

The predictive performance of all algorithms was assessed. In record linkage, the data is imbalanced, meaning that the two classes are not represented equally. Indeed, there are far more non-matches than matches. In such cases, standard accuracy is not a good measure of performance. Instead, precision and recall are commonly used for evaluating the linkage quality. We calculated these performance metrics based on the confusion matrix described in Table S1.

The precision is the proportion of classified matches that are true matches.

$$ precision=\frac{TP}{TP+ FP} $$

The recall is the proportion of true matches that have been classified correctly.

$$ recall=\frac{TP}{TP+ FN} $$

A good linkage algorithm will typically have values of precision and recall greater than 0.95 [29].

### Manual review

The role of the manual review is to verify whether the candidate matches identified by the linkage method are indeed true matches. In our study, the manual review was conducted by verifying the HGVS nomenclatures of *BRCA1/2* mutations, checking information on pedigrees, or verifying information on the original case report forms (CRFs) by contacting the recruiting center or the laboratory that performed the genetic test.

## Results

### Matching status assignment by PRL followed by manual review

**Fig. 2:**
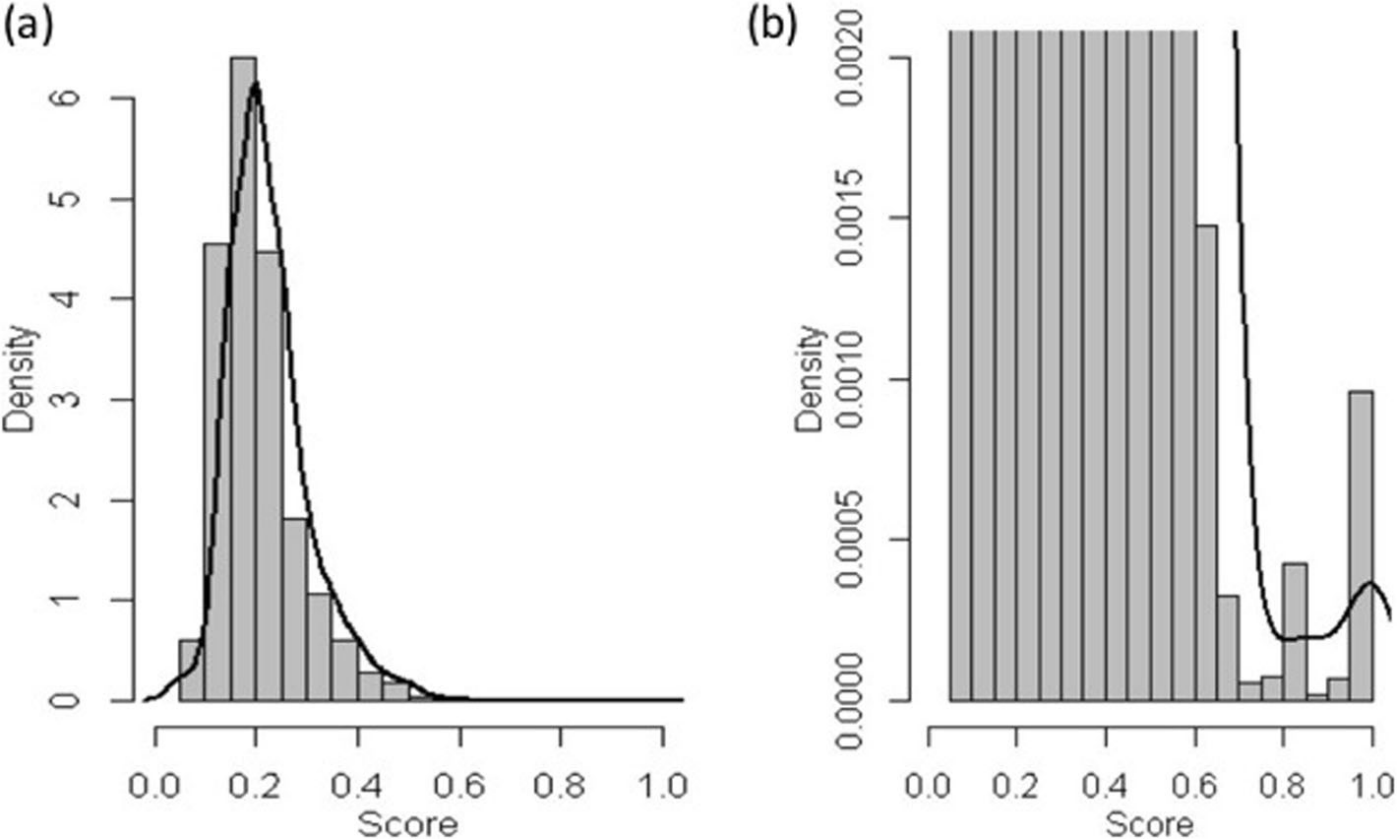
Score distribution of 15,653,232 record pairs in dataset 1. **a** Whole score distribution. **b** Zoom on the distribution for the highest scores

Up to September 2016, 4688 individuals had been enrolled in GEMO and 3339 in GENEPSO,. After data pre-processing, 15,653,232 record pairs were built as the Cartesian product of the two databases in dataset 1. The PRL score of each record pair was computed from Eq. 1 (see Methods). The empirical score distribution is given in Fig. 2 and Table S2. We observed a large peak of low scores, corresponding to a large number of non-matches, and a small peak of high scores, corresponding to a small number of matches. This bimodal distribution suggests that PRL worked as expected. After examining the distribution of PRL scores, we chose the threshold separating matches from non-matches at 0.6 and performed a step of manual reviews. 2,664 record pairs had a PRL score above 0.6, among which 751 pairs had a PRL score greater than 0.95. These 751 pairs were automatically considered as matches, because either all matching variables had a similarity score equal to 1, or one variable had a missing value and all others had a similarity of 1. The remaining 1913 potential matches were manually reviewed. In order to minimize the number of FN, we launched several rounds of searches to find out the record pairs whose PRL scores were below the threshold but were actual matches. We thus identified 11 additional true matches. All in all, 1257 pairs were classified as matches, and 15,651,938 pairs were classified as non-matches (Fig. 1a). Thirty-seven pairs whose matching status could not be determined by manual review were excluded.

### Selection of a supervised machine learning algorithm

We applied 5-fold cross-validation on the 15,653,195 record pairs that were labeled in the previous step. Within this cross-validation, we call A the training dataset (containing 12,522,556 record pairs) and B the test dataset (containing the remaining 3,130,639 record pairs). After blocking, each dataset A was randomly partitioned into a set Atrain containing 60% of the record pairs and a set Atest containing the remaining 40% of the record pairs (Table S3). The average predictive precision and recall of the Bernoulli model and of the six ML models, trained on Atrain and evaluated on Atest (Fig. 1b), are presented in Table 2. The six ML models outperformed the naive Bernoulli model, and their performance values, whether precision or recall, were all higher than 0.97, suggesting that they performed good linkage prediction. The RF algorithm showed the highest recall (0.9853) whereas the NNET algorithm showed the highest precision (0.9843). Here our objective was to have the highest possible recall, so as to identify as many true matches as possible. In addition, the RF algorithm required less tuning and was therefore more likely to generalize better. Therefore, we chose the RF algorithm as our ML algorithm.

**Table 2:** Mean performance for the ML algorithms trained on the Atrain dataset, evaluated on Atest

Models	Atest dataset			
	Recall		Precision	
	*M*	*SD*	*M*	*SD*
Bernoulli	0.01172	0.00079	0.01139	0.00096
CT	0.9841	0.016	0.9779	0.0059
Bagged trees	0.9809	0.012	0.9826	0.0080
AdaBoost	0.9839	0.011	0.9828	0.0075
RF	**0.9853**	0.011	0.9824	0.010
SVM	0.9821	0.017	0.9789	0.0068
NNET	0.9823	0.012	**0.9843**	0.0078

Six machine learning algorithms were tested: Classification Tree (CT), Bagged trees, AdaBoost, Random Forest (RF), Support Vector Machine (SVM) and Neural Network (NNET). *M* mean, *SD* standard deviation. The highest mean values among the different algorithms are highlighted in bold

### Evaluation of three linkage methods

**Fig. 3:**
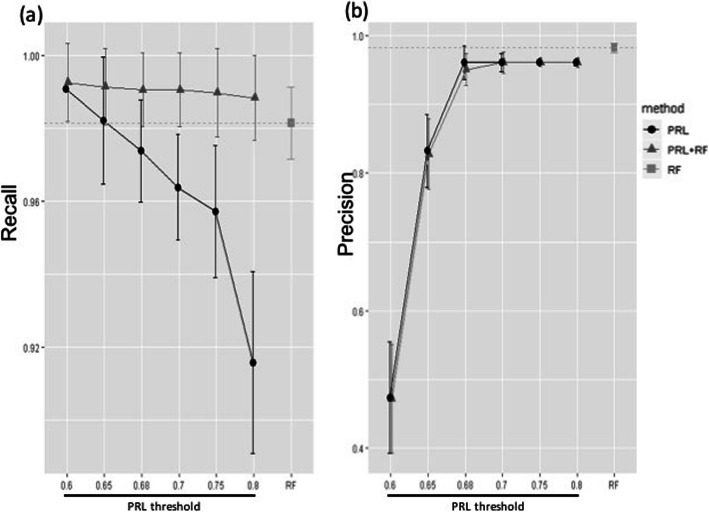
Performance of three linkage methods: PRL (Probabilistic Record Linkage), RF (Random Forest) and PRL + RF. PRL has thresholds varying from 0.6 to 0.8. **a** Comparison of their recalls. **b** Comparison of their precisions

In the 5-fold cross-validation step, the averaged performance of three linkage methods (PRL, RF, PRL + RF) was assessed on dataset B (Figs. 1c and 3). PRL and PRL + RF had thresholds varying from 0.6 to 0.8. As expected, increasing the threshold resulted in fewer FP, which led to an increase in precision, but more FN, which led to a decrease in recall. Across all thresholds, the PRL + RF method showed a higher recall than the RF (Fig. 3a). The recall of PRL decreased significantly with the threshold. The recall of RF was similar to that of PRL at threshold 0.65. RF achieved a higher precision than that of PRL and PRL + RF across all thresholds, while PRL and PRL + RF achieved similar precisions, which increased significantly with the threshold (Fig. 3b).

In conclusion, the PRL approach was very sensitive to the threshold and did not perform better than RF, except for the measure of recall at threshold 0.6, which, naturally, comes as the cost of a lower precision. Conversely, RF had a high precision but a modest sensitivity. PRL + RF had very high recall, and had a precision similar to that of PRL. Since the goal of our study was to minimize the number of FN, we chose the combined linkage method PRL + RF with the less conservative threshold of 0.6, which achieved the highest recall.

In order to train a final RF model (Fig. 1d) for PRL + RF, we applied blocking and imputation of missing data (see the missing rate of the six matching variables in Supplementary Data) on labeled 15,653,195 record pairs. We then randomly partitioned 107,599 pairs (after blocking) into a training set of 64,560 pairs and a test set of 43,034 pairs. The RF model still showed a good predictive performance (recall = 0.9916, precision = 0.9926). Thus, we used this RF model in PRL + RF method on updated databases.

### Linking records in updated GEMO and GENEPSO databases

**Fig. 4:**
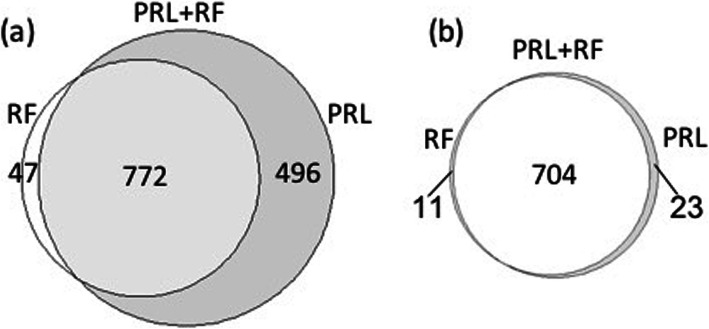
Comparison of candidate matches predicted by the RF and PRL models for the updated databases. **a** RF and PRL identified 819 and 1268 new candidate matches, respectively; 772 candidate matches were common to both approaches. **b** After manual review, PRL + RF led to the identification of 738 true matches, among which 727 were identified by PRL alone and 715 by RF alone. 704 true matches were identified by both approaches. 23 true matches were identified only by PRL, and 11 true matches were identified only by the RF model

As of December 2019, 1687 new *BRCA1/2* mutation carriers had been enrolled in GEMO and 1586 in GENEPSO. These updated GEMO and GENEPSO samples constitute dataset 2. The combined linkage method PRL + RF was applied on this dataset to identify the new true matches (Fig. 1e). Linkage was first performed by PRL and by RF separately. The RF model here was trained on a subset of dataset 1 (i.e. using GEMO and GENEPSO participants enrolled before September 2016) (Fig. 1d). RF and PRL predicted 819 and 1268 candidate matches, respectively (Fig. 4a). Besides the 772 matches that were common to these two linkage methods, RF had an additional 47 candidate matches and PRL had 496 additional candidates. Those 1315 (772 + 496 + 47) candidate matches were then manually reviewed. The PRL approach was correct for 57.3% (727 out of 1268) records; while the RF was correct for 87.3% (715 out of 819) records (Fig. 4b).

Finally, 738 of the 1315 candidate pairs suggested by the combined linkage method were true new matches. This is consistent with the precision achieved with a threshold of 0.6 on dataset 1 (Fig. 3b). The PRL + RF method identified 11 more true matches than PRL alone, corresponding to a gain of 1.5% (11/738), at a cost of manually examining 47 more candidate pairs. It also identified 23 more true matches than RF alone, corresponding to a gain of 3.1% (23/738), at a cost of manually examining 496 more candidate pairs (Fig. 4 and Table S4). This confirmed also that the PRL + RF method had a higher recall than PRL and RF alone.

To summarize, in December 2019, GEMO included 6375 participants and GENEPSO included 4925 participants, and our hybrid record linkage identified 1995 *BRCA1/2* mutation carriers from 1693 families that had been enrolled in both studies.

## Discussion

PRL has a lower computational cost but the linkage quality is impacted by the choice of the threshold on the likelihood score. Lower thresholds lead to more FP whereas higher thresholds lead to more FN. The ML approach reaches higher precision, requesting fewer manual reviews. However, the blocking step can lead to FN if the data contain errors in blocking variables. We found that the PRL + ML combined method, having the highest recall compared to either of the two methods alone, improves linkage by identifying more true matches, but at the cost of additional manual reviews.

In a context where manual review cost is to be capped and missing true matches is tolerated, the ML approach, which has a much higher precision to the expense of a lower recall, is an interesting option. Another possibility, which we expect from our results on dataset 1 (Fig. 3) to reach higher recall and higher precision, would be to use PRL + ML but with a higher threshold for PRL (such as 0.68 in our study). Here, our goal was to identify as many common participants as possible between the two studies, so as to facilitate research projects requiring both genetic and follow-up data. We therefore chose the PRL + ML approach with a relatively low threshold of 0.6 for PRL, so as to maximize recall.

We expect linkage performance to be related to the number of matching variables. Had more matching variables been shared between GEMO and GENEPSO, the most discriminating matching variables could have been identified using feature selection algorithms, resulting in a lower computational cost. Here, with 10 matching variables, such a strategy was not necessary. On the other hand, if too few matching variables had been available, one could expect ML models to have lower performance, giving the advantage to PRL.

Previously, Elfeky et al. [30] described a hybrid technique for record linkage, combining both supervised and unsupervised machine learning methods. Record pairs were assigned a matching or non-matching status through unsupervised clustering, and the resulting labeled data was then used as a training dataset for a supervised model [2]. However, this technique was not suitable here since two unsupervised machine learning methods (K-means and bagged k-means) showed independently poor performance (Supplementary Data, Table S5), probably due to our imbalanced data.

In this study, the two databases are limited in size. However, larger databases may be challenging for record linkage. In this case, the traditional blocking technique that we employed here is a first step towards reducing computational complexity. In addition, partitioning the data into a larger number of smaller blocks and processing them in parallel using our hybrid record linkage process could be used to maintain a reasonable computational time. Besides, In order to decrease the burden of manual review, we could aim to achieve a high precision instead of having high recall by choosing a higher PRL score threshold. Thus, the manual review could serve for linkage method tuning.

## Conclusions

**Fig. 5:**
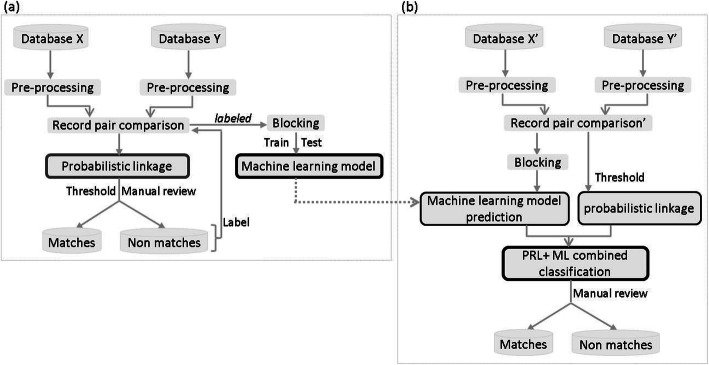
General overview of the hybrid record linkage process**. a** Probabilistic record linkage (PRL) followed by a stage of manual review is first applied to build a dataset allowing the construction of a supervised machine learning (ML) model. **b** The PRL + ML combined linkage is then used to classify the updated datasets (Record pair comparison from Database X’ and Database Y′). The ML model obtained in (**a**) is used (dotted arrow) for the prediction in (**b**)

In this paper, we propose a hybrid record linkage process which involves both PRL and ML approaches (Fig. 5). The hybrid process was developed using datasets from GEMO and GENEPSO which are two independent ongoing nation-wide epidemiological studies involving *BRCA1/2* mutation carriers. PRL and ML were combined to classify the record pairs into matches and non-matches, and the ML model was built on a training set labeled by using PRL followed by manual review.

GEMO and GENEPSO are ongoing studies and their respective databases are continuously updated. About 730 new subjects are included each year in GEMO, and 590 in GENEPSO. Hence, we will apply our hybrid approach on the updated versions of the two databases on a regularly basis, so as to identify new matches and increase the statistical power of research projects involving linked participants. Our hybrid record linkage process was driven by the need of a specific epidemiological question and may be generalizable to other epidemiological or translational studies involving other databases and registries.

## Supplementary Information

**Additional file 1:** **Table S1.** Confusion matrix. **Table S2.** Score distribution for all record pairs comparisons between GEMO and GENEPSO in dataset 1. **Table S3.** Size of each dataset A after blocking. **Table S4.** List of matches identified by either PRL or RF. **Table S5.** Performance of the unsupervised machine learning models.

## Data Availability

The GEMO dataset is included in the published article describing the GEMO resource [15]. The GENEPSO dataset is not publicly available but has been used in a number of studies [31–37]. Data are however available from the authors upon reasonable request.

## Abbreviations

BRCA1*: *BRCA1* mutational status
BRCA2*: *BRCA2* mutational status
CANSOP: Breast and ovarian cancer predisposition
CRFs: Case Report Forms
CT: Classification Tree
CTR*: Consultation center number
CV: Cross Validation
Dob*: Day of birth
FS: Fellegi and Sunter
FN: False Negative
FP: False Positive
GEMO: Genetic Modifiers of BRCA1 and BRCA2
GENDER*: Gender
GENEPSO: Prospective Study of BRCAx Gene Mutation
HGVS: Human Genome Variation Society
ML: Machine Learning
Mob*: Month of birth
MUT_HGVS*: Mutation description using the HGVS nomenclature
NUMFAM*: Family number of the consultation
NNET: Neural Network (with one hidden layer in this article)
PRL: Probabilistic Record Linkage
TN: True Negative
TP: True Positive
RF: Random Forest
SUJID*: Individual number in family
SVM: Support Vector Machine
Yob*: Year of born
