# Supplementary material for: A new hybrid record linkage process to make epidemiological databases interoperable: application to the GEMO and GENEPSO studies involving BRCA1 and BRCA2 mutation carriers
Source: BMC Med Res Methodol. 2021 Jul 29;21:155. doi: 10.1186/s12874-021-01299-6 (PMC8320036; doi:10.1186/s12874-021-01299-6)
Supplement: Supplementary file 1 — Additional file 1: Table S1. Confusion matrix. Table S2. Score distribution for all record pairs comparisons between GEMO and GENEPSO in dataset 1. Table S3. Size of each dataset A after blocking. Table S4. List of matches identified by either PRL or RF. Table S5. Performance of the unsupervised machine learning models. [file 12874_2021_1299_MOESM1_ESM.docx]

**Supplementary Data**

**Materials and Methods**

**Jaro-Winkler similarity.** The Jaro-Winkler similarity[1] was built from the Jaro similarity as follows:

| $sim_{JW}\left( x,y \right)=sim_{J}\left( x,y \right)+lp\left( 1-sim_{J}\left( x,y \right) \right)$ |  |
| --- | --- |

where $l$ is the length of common prefix at the start of the string up to a maximum of four characters and $p$ is a constant scaling factor for how much the score is adjusted upwards for having common prefixes.p $p$ should not exceed $0.25$, otherwise the distance can become larger than 1. The standard value for this constant in Winkler’s work[2] is $p=0.1$.

The Jaro similarity[3] is defined as:

| $sim_{J}\left( x,y \right)=\frac{1}{3}\left( \frac{c}{\vert x\vert}+ \frac{c}{\vert y\vert}+ \frac{c-t}{c} \right)$ |  |
| --- | --- |

where $|x|$ is the length of the string, $c$ is the number of matching characters (characters that are the same) that are in common within half the length of the longer string, and $t$ is the number of transpositions (two adjacent characters that are swapped in the two strings, such as ‘de’ and ‘ed’).

**Binary similarity.** The Binary similarity (*i.e.* extract agreement) is defined as:

| $sim_{B}\left( x,y \right)=\left\{ \begin{matrix} 1 & if x=y, \\ 0 & otherwise. \end{matrix} \right.$ |  |
| --- | --- |

An example is shown in Table 2 where the first and the second row correspond to the vector $x$ and $y$ respectively and the third row is the value of the similarity vector $s\left( x,y \right)$.

**K-means clustering.** K-means clustering[4] is an iterative algorithm that partitions n observations into k clusters in which each observation belongs to the cluster with the nearest mean (or centroid). At each step, it calculates the centroids of the k clusters, and reassigns each data point to the nearest cluster (*i.e.* the one with closest centroid). K-means is fast and flexible.

**Bagged K-means clustering.** Bootstrap Aggregating (Bagged)[5] is an ensemble method for clustering. The idea is to stabilize K-means, which is a stochastic algorithm and may not return the same clusters each time it is run, by repeatedly running it and combining the results by hierarchical clustering.

**Classification Tree (CT).** CART (Classification and Regression Tree)[6] can build classification (categorical outcome) or regression (continuous outcome) models represented as a binary tree. At each node, the record pairs are split into two subsets (nodes) based on thresholding the value of the identifiers describing them (here, the similarity score for each identifier). GINI impurity measure is used for selecting identifiers and best threshold[7]. Once a tree is built, pruning is applied to select a tree size that minimizes the cross-validated error, in order to reduce the overfitting and complexity.

**Bagged trees.** Bootstrap aggregating (Bagging)[8] is an ensemble algorithm. Used with classification trees, it improves the stability of a model by reducing variance and thus avoiding overfitting. The models are built independently and parallelly. The predictions from these models are aggregate and make a majority voting to determine the final class.

**AdaBoost.** Boosting is an ensemble technique that attempts to create a strong model from a number of weak models, typically classification trees. AdaBoost (Adaptive Boosting)[9] is one of the most widely used boosting algorithm. AdaBoost is a sequential ensemble where each model is built based on correcting the misclassifications of previous model, and it involves the weighted majority voting of sequential models. Each sequential model is built by assigning greater weights to misclassified data points of the previous model.

**Random Forest (RF).** The RF algorithm[10] is also an ensemble of unpruned classification trees. It is an improvement over bagged classification trees. Two components of randomness are introduced into the construction of the individual tree: each tree grows on an independent dataset, which is a bootstrap sample from the training data; at each node, a few identifiers are randomly selected out of all identifiers, and the best split is determined among these selected identifiers only. Prediction is made by aggregating (majority vote for classification) the predictions of the ensemble.

**Support Vector Machine (SVM)**. A SVM[11] separate positive and negative examples with a hyperplane, constructed in such a way as to have maximum margin (*i.e.* distance from the training points to the hyperplane), since the larger the margin, the lower the generalization error of the model[12]. Support vectors are the data points that lie closest to the hyperplane. The classification of new data is based on which side of the hyperplane the data point falls. SVMs can perform non-linear classification by transforming the data using a kernel function such as Gaussian Radial Basis Function (RBF), which is commonly used in SVM classification.

**Neural Network (NNET)** Single-layer neural network[13] can be viewed as three layers of connected nodes (called neurons or units). The input layer contains the nodes representing identifiers describing the data (here, the similarities $s_{i}$ for each identifier). In the case of a binary classification (here, matches vs. non-matches), the output layer contains a single node. A ’hidden’ layer between input and output layers contains a number of nodes, to be tuned during training. The layer is known as “hidden”, since it is not visible as a network output. Every node in the hidden layer is connected to each node in input layer and each node in output layer. Each connection has an associated weight, which is learned during training. Each node applies an activation function to the weighted sum of its inputs plus a constant (called the bias). The activation function (typically a logistic/sigmoid function) introduces non-linearities. The output of the network is interpreted as the probability (between 0 and 1) of belonging to the positive class (here, matches). A threshold of 0.5 is used to separate the two classes.

The record pair comparisons and PRL were implemented using the RecordLinkage R package[14]. The e1071[15], caret[16], mice[17], ada[18], ipred[19], randomForest[20], nnet[21] packages in R were used for the implementation of the machine learning methods.

**Hybrid record linkage process - Step 2**

In the 5-fold cross validation, all record pairs were partitioned into five equal size groups. Of the five groups, a single group (dataset B) was retained for comparing the predictive performance of the three linkage methods. The remaining four groups (dataset A) were used to select an optimal ML algorithm.

For each round of cross validation, we performed the following steps:

(1) we applied blocking on dataset A to reduce the number of possible record pair comparisons. Missing data in blocking variables (BRCA1, BRCA2, GENDER and Yob) were tolerated here. After blocking, missing data was imputed.

(2) we randomly partitioned dataset A into two sets: 60% of record pairs were used as training dataset (Atrain) on which we trained ML models, and the remaining 40% were used as a test dataset (Atest) on which we evaluated the predictive performance of the trained ML models. We employed six broadly used ML algorithms (CT, Bagged trees, AdaBoost, RF, SVM and NNET). In order to determine the optimal parameters for each of these ML algorithms, we carried out an inner 5-fold cross validation on each Atrain when building a ML model. We also built a naive baseline Bernoulli model on which other ML models can be compared to. We calculated the average performances of 6 ML algorithms on dataset A from five rounds and chose an optimal ML algorithm.

(3) The chosen ML model built from dataset A was used when applying ML and PRL+ML linkage methods on dataset B. We set the PRL score thresholds varying from 0.6 to 0.8 in the PRL and PRL+ML methods, since the number of FP and FN was very sensitive to the threshold. Finally, we compared the average predictive performance of three linkage methods on dataset B from five rounds and chose the method that best meets our needs. As the goal of our study is to minimize the number of FN, we used recall as a selection criterion.

**Results**

## Missing rate of matching variables

Before the imputation of missing data, we checked the missing rate for the six matching variables that would be used in ML. CTR missing rate = 0, NUMFAM missing rate = 0.048%, SUJID missing rate = 0.063%, MUT_HGVS missing rate = 2.5%, Mob missing rate = 0.14% and Dob missing rate = 0.14%.

### Unsupervised classification model

We used K-means and Bagged clustering to separate the record pairs into two clusters: matches and non-matches. Their performances are reported on Table S3. Although the recall was quite satisfying, both of them showed too many FP (almost half of the total record pairs) inducing a low precision and F-measure. Hence we did not proceed further with unsupervised classification.

|  | | True match status | |
| --- | --- | --- | --- |
|  |  | Non-Matches | Matches |
| Linkage | Non-Matches | TN (True Negative) | FN (False Negative) |
|  | Matches | FP (False Positive) | TP (True Positive) |

**Table S1. Confusion matrix.** The performance metrics are calculated from the confusion matrix.

| Score range | Frequency |
| --- | --- |
| [0,0.05] | 184865 |
| (0.05,1] | 278469 |
| (0.1,0.15] | 3568224 |
| (0.15,0.2] | 5012178 |
| (0.2,0.25] | 3502947 |
| (0.25,0.3] | 1415677 |
| (0.3,0.35] | 833321 |
| (0.35,0.4] | 470950 |
| (0.4,0.45] | 210737 |
| (0.45,0.5] | 142424 |
| (0.5,0.55] | 25613 |
| (0.55,0.6] | 8502 |
| (0.6,0.65] | 1157 |
| (0.65,0.7] | 253 |
| (0.7,0.75] | 44 |
| (0.75,0.8] | 56 |
| (0.8,0.85] | 332 |
| (0.85,0.9] | 16 |
| (0.9,0.95] | 55 |
| (0.95,1] | 751 |

Table S2. Score distribution for all record pairs comparisons between GEMO and GENEPSO in dataset 1. The score is between 0 and 1.

| Dataset | N. record pairs | Dataset | N. record pairs |
| --- | --- | --- | --- |
| A1 | 87618 | A1train | 52571 |
|  |  | A1test | 35047 |
| A2 | 85351 | A2train | 51211 |
|  |  | A2test | 34140 |
| A3 | 83657 | A3train | 50195 |
|  |  | A3test | 33462 |
| A4 | 87020 | A4train | 52212 |
|  |  | A4test | 34808 |
| A5 | 86750 | A5train | 52050 |
|  |  | A5test | 34700 |

**Table S3. Size of each dataset A after blocking.** Each dataset A was randomly patitioned into two sets: the training dataset (60%) Atrain and the test dataset (40%) Atest.

| A. Example of 4 matches identified by PRL: | | | | | | | | | | | | | |
| --- | --- | --- | --- | --- | --- | --- | --- | --- | --- | --- | --- | --- | --- |
|  | BRCA1 | BRCA2 | CTR | GENDER | NUMFAM | SUJID | MUT_HGVS | Yob | Mob | Dob | Weight | |  |
| pair 1: |  |  |  |  |  |  |  |  |  |  |  | | |
| GEMO | 1 | NA | 01 | 2 | 23019 | 0003 | c.3947_3950del | **1992** | **06** | 02 | 0.7804626 | | |
| GENEPSO | 1 | NA | 01 | 2 | 23019 | 0003 | c.3947_3950del | **2017** | **10** | 02 |  | | |
| pair 2: |  |  |  |  |  |  |  |  |  |  |  | | |
| GEMO | NA | 1 | 01 | 2 | 23636 | 0001 | **c.275_278dup** | **1993** | **08** | **18** | 0.6910272 | | |
| GENEPSO | NA | 1 | 01 | 2 | 23636 | 0001 | **c.275-278dup** | **2017** | **07** | **03** |  | | |
| pair 3: |  |  |  |  |  |  |  |  |  |  |  | | |
| GEMO | 1 | NA | 01 | 2 | **7790** | 0003 | **c.3841C>T** | 1987 | 02 | 03 | 0.7428885 | | |
| GENEPSO | 1 | NA | 01 | 2 | **07790** | 0003 | **c.3481C>T** | 1987 | 02 | 03 |  | | |
| pair 4: |  |  |  |  |  |  |  |  |  |  |  | | |
| GEMO | NA | 1 | 01 | 2 | 21532 | 0001 | c.9672dup | **1963** | **06** | **13** | 0.6971889 | | |
| GENEPSO | NA | 1 | 01 | 2 | 21532 | 0001 | c.9672dup | **2017** | **07** | **03** |  | | |
|  | | | | | | | | | | | | | |
| B. Example of 2 matches identified by RF model: | | | | | | | | | | | | | |
|  | BRCA1 | BRCA2 | CTR | GENDER | NUMFAM | SUJID | MUT_HGVS | Yob | Mob | Dob | |  | |
| pair 1’: |  |  |  |  |  |  |  |  |  |  | |  | |
| GEMO | 1 | **NA** | 22 | 2 | **N05012** | **IV-5** | c.4868C>G | 1983 | 11 | 07 | |  | |
| GENEPSO | 1 | **0** | 22 | 2 | **05012** | **4001** | c.4868C>G | 1983 | 11 | 07 | |  | |
| pair 2’: |  |  |  |  |  |  |  |  |  |  | |  | |
| GEMO | **NA** | 1 | **58** | 1 | **LR09045** | **3002** | c.6079dup | 1943 | 05 | 10 | |  | |
| GENEPSO | **0** | 1 | **22** | 1 | **09045** | **3001** | c.6079dup | 1943 | 05 | 10 | |  | |

Table S4. List of matches identified by either PRL or RF. A. Example of 4 matches identified by PRL. B. Example of 2 matches identified by RF model. The bold text represents the difference between two records. There are ten shared identifiers between GEMO and GENEPSO: *BRCA1* mutational status (BRCA1), *BRCA2* mutational status (BRCA2), mutation description using the HGVS nomenclature (MUT_HGVS), gender (GENDER), consultation center number (CTR), family number of the consultation (NUMFAM), individual number in family (SUJID), year of birth (Yob), month of birth (Mob) and day of birth (Dob).

| **K-means** (k=2) | Reference | |  | **Bagged K-means** | Reference | |
| --- | --- | --- | --- | --- | --- | --- |
| Prediction | 0 | 1 |  | Prediction | 0 | 1 |
| 0 | 7543508 | 70 |  | 0 | 7688718 | 3 |
| 1 | 8108430 | 1187 |  | 1 | 7963220 | 1254 |

| Model | Balanced Accuracy | Recall | Precision | F-measure |
| --- | --- | --- | --- | --- |
| K-means | 0.71313 | 0.94431 | 1.464e-04 | 2.927e-04 |
| Bagged K-means | 0.74442 | 0.99761 | 1.574e-04 | 3.148e-04 |

**Table S5. Performance of the unsupervised machine learning models.**

# References

1. Yancey WE. Evaluating string comparator performance for record linkage. Statistics. 2005;5.

2. Winkler WE. String Comparator Metrics and Enhanced Decision Rules in the Fellegi-Sunter Model of Record Linkage. 1990.

3. Jaro MA. Probabilistic linkage of large public health data files. Statistics in medicine. 1995;14:491–498.

4. Hamerly G, Elkan C. Alternatives to the k-means algorithm that ﬁnd better clusterings. :8.

5. Leisch F. Bagged clustering. 1999.

6. Breiman L. Classification and regression trees. Routledge; 2017.

7. Therneau TM, Atkinson EJ. An introduction to recursive partitioning using the RPART routines. Technical Report 61. URL http://www. mayo. edu/hsr/techrpt/61. pdf; 1997.

8. Quinlan JR. Bagging, boosting, and C4. 5. In: AAAI/IAAI, Vol. 1. 1996. p. 725–730.

9. Freund Y, Schapire RE. A decision-theoretic generalization of on-line learning and an application to boosting. Journal of computer and system sciences. 1997;55:119–139.

10. Breiman L. Random forests. Machine learning. 2001;45:5–32.

11. Cortes C, Vapnik V. Support-vector networks. Machine learning. 1995;20:273–297.

12. Friedman J, Hastie T, Tibshirani R. The elements of statistical learning. Springer series in statistics New York, NY, USA:; 2001.

13. Auer P, Burgsteiner H, Maass W. A learning rule for very simple universal approximators consisting of a single layer of perceptrons. Neural networks. 2008;21:786–795.

14. Sariyar M, Borg A. The RecordLinkage Package: Detecting Errors in Data. R journal. 2010;2.

15. Meyer D. Support Vector Machines: The Interface to libsvm pacakge e1071. Technische Universität Wien, Austria. 2012.

16. Kuhn M. Caret package. Journal of statistical software. 2008;28:1–26.

17. van Buuren S, Groothuis-Oudshoorn K, van Buuren MS, Suggests VIM. Package ‘mice.’ Computer software] Retrieved from: http://cran r-project org/web/packages/mice/mice pdf. 2015.

18. Culp M, Johnson K, Michailidis G. ada: An r package for stochastic boosting. Journal of Statistical Software. 2006;17:9.

19. Peters A, Hothorn T, Hothorn MT. Package ‘ipred.’ 08-7 The R Foundation for Statistical Computing. 2009.

20. Breiman L, Cutler A, Liaw A, Wiener M. Package randomForest. Software available at: http://stat-www berkeley edu/users/breiman/RandomForests. 2011.

21. Ripley B, Venables W, Ripley MB. Package ‘nnet.’ R package version. 2016;:7–3.
